# Client and Practitioner Insights into Treatment for Women Convicted of Child Sexual Abuse: A Qualitative Study Informing Gender-Responsive Practice

**DOI:** 10.1177/10790632261470828

**Published:** 2026-07-22

**Authors:** Bricklyn Priebe, Nadine McKillop, Susan Rayment-McHugh, Larissa S. Christensen

**Affiliations:** 1 Sexual Violence Research and Prevention Unit, School of Law and Society, 5333 University of the Sunshine Coast, Maroochydore DC, QLD, Australia

**Keywords:** gender responsivity, female-perpetrated sexual offending, therapy, intervention, user voice

## Abstract

Gender-responsive programming has emerged as a pivotal force behind improved therapeutic outcomes for women in the criminal justice system. Enhancing relevance of treatment provided to women sentenced for child sexual abuse (CSA) is necessary given the proposed gender-based pathways and motivations linked with this crime. This research incorporates the views of women sentenced for CSA (*n* = 18) and practitioners (*n* = 25) and aims to determine the key treatment targets for women sentenced for CSA, and how these might be similar or different to the needs of men sentenced for this offending. Results indicate that despite some overlap of treatment targets, there are gendered nuances in how some core topics should be addressed when delivering offense-specific correctional programming to women. Five key targets emerged that indicate notable differences warranting tailored intervention, including: the effects of past trauma, relationship toxicity, (un)healthy sexuality, lowered self-concepts, and parenting challenges. These nuances align with strengths-based and trauma-informed approaches advocated for this population, helping to guide future program development and implementation. Recommendations for practice are included.

## Introduction

Meta-analytic research indicates that women comprise approximately 2-5% of detected perpetrators in sexual offense cases, highlighting their small but significant representation within recorded data (Cortoni et al., 2017). Other studies, however, suggest that women may be responsible for up to one in five cases (e.g., McLeod, 2015), thus showing variability in prevalence estimates depending on data sources, definitions, and reporting practices, and likely reflecting ongoing underreporting of abuse perpetrated by women. In Australia, where the present study is primarily situated, the number of females proceeded against by police for a principal offense of sexual assault increased by 207.66% from 2008-2023 (*n* = 222, 2.3 per 100,000 – *n* = 683, 5.8 per 100,000; Australian Bureau of Statistics, 2024). While this rise in recorded offenses might reflect increased prosecution and reduced diversion rather than an actual increase in offending behavior, it nonetheless highlights the growing recognition of women as potential perpetrators of sexual abuse. The harms caused are also understood to be significant, long-lasting, and equal the harms caused by men (Cortoni, 2018; Gerke et al., 2023). Consequently, there is an increasing need for responsive interventions for women who perpetrate CSA to reduce their likelihood of causing future harm, recognizing that reoffending may include sexual, violent, or other general offenses (Hanson et al., 2025).

Gender responsivity in correctional practice involves active consideration and purposeful adjustments to program design, delivery, and implementation. This includes assessment of “site selection, staff selection, program development, and program content and materials” to ensure they are reflective of the female experience and gendered pathways in to — and out of — crime (Covington, 2008, p. 377; Gower et al., 2024). Demand for responsive correctional programming for women sentenced for CSA stems from research demonstrating gender-specific offense pathways. For example, women involved in CSA often report higher personal victimization rates (Augarde & Rydon-Grange, 2022), including domestic violence within the family home (McLeod, 2015; Neofytou, 2022), and they are more likely to offend in the presence of an accomplice, mostly a male partner (Augarde & Rydon-Grange, 2022; Williams et al., 2019). However, co-offending among women is not limited to mixed-gender partnerships, with all-female co-offending groups representing a small proportion of known cases (e.g., Budd et al., 2017; Vandiver, 2006). Gendered nuances in the motivations for involvement with CSA are also apparent; women are more likely to offend due to fear of — or actual — harm from a male co-offender (Gannon et al., 2012) and may be motivated to participate in the abuse to prevent their own victimization (Beech et al., 2009). Like men, women are also reportedly motivated to perpetrate CSA to fill the void of “intimacy and warmth” in their adult relationships (Gannon et al., 2012, p. 219). In contrast to men, though, this often leads to co-offending with a male perpetrator in an effort to maintain intimacy in their relationship (Augarde & Rydon-Grange, 2022), rather than seeking intimacy from the child. In these instances, the abuse is more often perpetrated against the woman’s own child/ren (Wijkman et al., 2011). While deviant sexual interests are a factor in the offending of some women, these are less common when compared with men (Sousa et al., 2024). Nevertheless, limited research has identified cases involving paraphilic arousal and, more rarely, sexual sadism among women who perpetrate CSA (Chopin et al., 2024; Pflugradt & Allen, 2012), indicating that deviant sexual interests may contribute to offending for some women.

Gendered treatment frameworks for women emphasize the need for strength-based and trauma-informed approaches (Cortoni, 2018, 2024; Pflugradt & Allen, 2019; Pflugradt et al., 2018, 2022), and consistent with the responsivity principle (see: Andrews & Bonta, 2010), programs that attend to the gender-specific treatment needs of women have been associated with improved engagement and program completion (e.g., Gobeil et al., 2016; Wanamaker et al., 2018). Formalized sexual offending treatment programs (SOTPs) for women have been developed in some countries, such as Canada, the United States, and the United Kingdom, however, few comprehensive evaluations have examined their longer-term effectiveness in reducing sexual recidivism, and larger sample sizes are needed to determine measurable outcomes (Sousa et al., 2024; Wanamaker et al., 2018). Indeed, there is still much to learn about the treatment needs, composition, and efficacy of treatment for this population. For example, recent meta-analytic research suggests that women who perpetrate sexual offenses typically present as a low-risk (of sexual recidivism), but high-need population (Hanson et al., 2025), pointing to the relevance of treatment outcomes beyond sexual recidivism, and highlighting the ongoing importance of careful individualized assessment to ensure treatment intensity and targets are appropriate for each client (Carr & Willis, 2021). However, despite significant advancements in research and practice indicating these elevated needs, further work is required to explore the gendered nuances in *how* these needs should be addressed in practice (e.g., Cortoni & Gannon, 2013; Sousa et al., 2024). Thus, in the absence of formal and sophisticated program evaluation and application to date, continued research is necessary to refine key treatment considerations and operationalize gender-informed intervention priorities, building on emerging conceptual and assessment frameworks that support gender-responsive practice (Cortoni, 2018, 2024; Pflugradt et al., 2022).

Advancing understanding of what works in treatment should encompass the perspectives of “the practiced-upon” and “the practitioner”, yet these perspectives are often ignored or underutilized (Bullock & Bunce, 2018; Osburn et al., 2011, p. 218). This presents a missed opportunity, as incorporation of these perspectives has been found to be intrinsically connected to more effective program outcomes; incorporation of the user-voice also helps to facilitate greater alignment between programs provided to clients and those actually desired by clients (Weaver & McColloch, 2012). Practitioners offer insight “from the trenches” (Tewksbury & Mustaine, 2012, p. 427), with knowledge and expertise on current frameworks and guidelines relevant to treatment and management of those who offend. Justice involved women are experts by “lived experience” (Visser et al., 2021, p. 1) and are able to offer suggestions about program content and preferences based on these experiences (Priebe et al., 2024b). However, their perspectives, and those of practitioners, have not previously been sought within the Australian context, despite increasing recognition of the value of lived-experience insights in shaping effective policy and practice (McCartan et al., 2019). Engaging directly with these voices is essential to shaping effective, gender-responsive interventions, particularly in Australia, where empirical research and formal program development in this area remain extremely limited. Hence, this study aims to determine the relevant treatment needs of women sentenced for CSA, utilizing these voices to gain a deeper understanding of program components and key responsivity considerations.

## The Present Study

This study is part of a larger research project focused on key treatment considerations for women who perpetrate CSA. Findings from this research have so far established a demand for SOTPs for women who commit CSA (Priebe et al., 2024a), have provided insight into how treatment is best delivered, and identified potential barriers for implementation (Priebe et al., 2024b, 2025). Drawing on the perspectives of Australian justice-involved women alongside practitioners with experience across several international jurisdictions, the present study both complements and extends these findings by identifying what treatment should address (i.e., program content). The aim is to inform tailored interventions for women convicted for CSA within the Australian context, where gender-responsive programs remain underdeveloped, however, key insights are likely transferable to comparable settings internationally.

### Participants

The present study involved two sample populations: (1) practitioners currently employed in either correctional settings (community and custodial) or tasked with providing therapeutic services in non-government affiliated community settings, and (2) women (herein referred to as justice-involved women) currently serving a custodial or community-based correctional order for CSA in Australia.

#### Practitioners

Practitioners were invited to participate in this study if they were over the age of 18, had English-language proficiency, and worked in a capacity to design, deliver, and/or manage interventions for female adults/adolescents involved with CSA perpetration. To broaden understanding of current design, implementation, and management considerations from settings where gender-responsive programs are more established, practitioners were recruited from multiple jurisdictions: Canada, the United Kingdom, New Zealand, and Europe, alongside Australia; most were female (*n* = 22, 88%). The majority worked in non-government therapeutic positions (*n* = 14, 56%), such as in private practice or within non-profit organizations; the remainder were employed by corrective service departments in three Australian jurisdictions (*n* = 11, 44%). To ensure the anonymity of participants, identification of specific jurisdictional locators was excluded.

A small minority of participants recruited from non-government therapeutic settings (*n* = 3, 21.4%) had practice-based experience working exclusively with adolescent girls engaged in harmful sexual behaviors; confirmation was sought from these participants to determine the perceived applicability of their suggestions for treatment of adult women, and their input was incorporated where relevant to the broader sample, serving as an additional form of triangulation to enhance analytic confidence.

#### Justice-Involved Women

A total of 18 participants were recruited from correctional services across three Australian jurisdictions. Participants were all over the age of 18, had English-language proficiency, were born – and continued to identify – as female, and were currently serving a sentence for CSA perpetration. Participants were primarily serving custodial sentences (83.3% *n* = 15), one participant was on a community sentence, and two participants were on community sentences (i.e., parole) following release from custody. Almost all participants were born in Australia (*n* = 16, 88.8%) although none identified as First Nations Australians. Participants had an average age of 42 (range = 26 to 69 years, *SD =* 10.18). The length of correctional sentence ranged from 18 months to 28 years (*M* = 10.12, *SD* = 6.54). Almost all self-reported that their conviction for CSA was their first involvement with the criminal justice system (*n* = 17, 94.4%).

### Measures and Procedure

Ethics approval for this research was granted by the University of the Sunshine Coast Research Ethics Committee (No. S211536/S221688). Further departmental research approvals were granted from three respective Corrective Services departments within Australia, which permitted the involvement of justice-involved women and correctional practitioners.

#### Interviews With Practitioners

A purposive sampling method was used for non-government-affiliated practitioners. These participants were emailed directly by the first author following a database search of relevant organizations and private practitioners, as well as existing contacts in the field (Campbell et al., 2020). Correctional practitioners were forwarded the recruitment text via an internal departmental research liaison. Individuals who elected to participate did so via an anonymous online interview sign-up page, and were subsequently interviewed by the first author. Interviews followed a semi-structured format and were approximately 45 minutes each (*M* = 46.9 min; *SD* = 10.5). Interviews were conducted online via videoconference (*n* = 23, 92%) and two occurred via telephone. Interview questions focused on what programs/services are currently provided, as well as suggestions for how programs for women sentenced for CSA could be more gender-responsive (e.g., what are the current strengths/limitations of programs offered to women who have perpetrated CSA where you work? If you could design a program specifically for women sentenced for CSA, what would it include?).

#### Interviews With Justice-Involved Women

Interviews were facilitated with the assistance of correctional departments from three Australian jurisdictions and involved an appointed liaison in each state/territory explaining the project, and providing a written research information sheet, to all justice-involved women meeting the study eligibility criteria. Following an expression of interest in participating in the research, the liaison then contacted the first author, on behalf of the participant, to schedule a suitable interview time and location.

The majority of interviews occurred via videoconference (*n* = 8, 44.4%) or in-person (*n* = 6, 33.3%), and some (*n* = 4, 22.2%) were completed via telephone. Interviews with justice-involved women followed a semi-structured format and were approximately 45 minutes each (*M* = 41 min; *SD* = 11.5). Interviews focused on the past experiences of correctional programs and participant suggestions for future program offerings (e.g., If you could design a program for women sentenced for crimes similar to you, what would that program look like?).

Interviews with both sample populations concluded following saturation; while this is a subjective determination, it was guided by data repetition across sample populations (Braun & Clarke, 2021).

### Analytic Strategy

The first author conducted all interviews; all interviews were recorded and then transcribed verbatim. A total of 43 interview transcripts were examined.

A reflexive thematic analysis (Braun & Clarke, 2006, 2021) was followed to examine interview data. Practitioner interviews (*n* = 25) involved a combined inductive-deductive analysis (Fereday & Muir-Cochrane, 2006). No predetermined theories or codes were used, however, the researcher specifically looked for instances where participants provided cross-gender comparisons (i.e., ‘This is different to men’), consequently providing a more detailed analysis – and nuanced understanding – of the data (Vaismoradi et al., 2013). The researcher then used a latent analysis approach to examine interviews involving justice-involved women (*n* = 18) to identify key themes, based on first-hand accounts.

The six-step process of thematic analysis (Braun & Clarke, 2006, 2021) was followed to examine interviews: (1) the first author immersed themselves in the interview transcripts; (2) codes were formed; (3) more nuanced themes within the dataset subsequently developed; (4) a sample of interview data was then shared and reviewed by all members of the research team to facilitate greater objectivity in the analysis of qualitative results; (5) the research team met on several occasions to discuss findings and reach consensus on themes and theme titles; and (6) a sample of quotations most representative of the themes were identified and data from both participant groups were merged and results were written.

## Results

Five key themes emerged from the interview data related to gender-responsive treatment considerations for women sentenced for CSA: (1) the effects of past trauma; (2) relationship toxicity; (3) (un) healthy sexuality; (4) lowered self-concepts; and (5) parenting challenges. The findings below focus on explaining key responsivity considerations when designing programs for women, from the perspective of practitioners (denoted as ‘PP’), as well as women currently serving a sentence for CSA in Australia (denoted as ‘JIP’).

### Theme 1: The Effects of Past Trauma

Practitioners indicated that the involvement of women in CSA is less a result of sexual interest, and more likely connected to complex trauma and relationship distortions instead. All practitioners in the interview sample explained that these women have often experienced significant trauma histories stemming from childhood, including past sexual and physical victimization and witnessing domestic violence. They explained that this largely shapes the offense-pathways for women, and even their motivations for involvement in this crime, which is often distinct when compared with men who offend. For example, “More of women’s offending is based around trauma backgrounds as opposed to that really antisocial and pro-criminal attitude, which is sometimes more relevant for men” (PP3).

Many noted that addressing past and accumulated trauma is central to the treatment needs of women who have perpetrated CSA, and that this is often criminogenic and therefore a necessary treatment target to prevent re-offending. Practitioners emphasized the importance of acknowledging past experiences of victimization before moving on to forensic treatment involving the women’s own offending, as this will help to improve treatment readiness and program retention:

For women, there needs to be a start with them receiving empathy and understanding for their own victimization. Cuz I don't think you're ever going to help them develop empathy and understanding for the person that they've harmed until they've received some support themselves (PP13).

Almost all practitioners noted that the short- and long-term toll of trauma experiences has a significant impact on the self-identity of women, and suggested that this should be the primary focus during treatment, rather than in-depth discussions about the crime event itself.

The need to address past victimization histories during treatment was also identified through first-hand accounts of women sentenced for CSA. The majority of the justice-involved women in this study identified significant trauma experiences and questioned how these may have influenced their offense-pathways and their involvement in unhealthy relationships as adults. For example, “I have really bad boundaries around men, being molested as a child by my biological father, my uncle who was married into the family, by a next-door neighbor, my dad, my grandfather, and my first partner” (JIP5).

Some of the justice-involved women believed that a breakdown of their family unit during childhood had relevance to explaining their pathways into crime; they reported struggles with mental health, issues with self-esteem and feelings of rejection, housing instability, and vicarious conflict, as having a significant impact. Largely, the women in this study expressed a desire to learn more about their pathways into offending; many wanted guidance for questions about “why I did what I did” (JIP1), and were hopeful that a program would allow them support in exploring their often-turbulent backgrounds in a safe space. In addition to a desire for more support with understanding the effects of past trauma on offending pathways, the justice-involved participants in this study also identified a need for strategies to cope with the ongoing effects of trauma. Some reported that their trauma responses caused them to shut down when confronted with triggers from their past: “I suffer from PTSD so certain things trigger memories and I just shut down… I’ll just freeze, and shrink, and wait for someone to yell at me” (JIP10).

Consistent with the views of practitioners regarding the need for trauma-informed interventions for this population, many of the justice-involved women identified that flashbacks were an ongoing concern, contributing to declined mental health, and/or leading them to unhealthy coping mechanisms, such as substance use, to “forget” (JIP1) or “numb the pain” (JIP5).

### Theme 2: Relationship Toxicity

Connected to the issue of trauma for women is the experience of ongoing exploitation and abuse in domestic relationships, often for a significant duration and high-level frequency. Of significance, almost all practitioners indicated that unhealthy intimate relationships are directly connected to the offending trajectories of many women who perpetrate CSA. One participant said, “I think relationships in particular for females…it’s true of males too, but I think in particular for females, relationship issues are absolutely central to the problem” (PP15).

The influence of male partners, who are often co-offenders, is a gender-specific characteristic of women’s involvement in this crime. Some practitioners stated that this impacts female involvement in CSA directly in terms of coercion involving threats or actual violence. However, the majority of practitioners noted that pressure from male partners was less blatant, and instead more of a reflection of relationship toxicity and a desire for women to want to please their partners to help maintain the relationship:

Females are often looking for…that relationship component in terms of intimacy, or they have been abused within that relationship and that's why she's doing it, so the normal sort of distortions that we might assume are relevant for men can be quite different with a female (PP14).

In terms of treatment focus, practitioners highlighted that there may be a need to counsel women on independence to minimize their involvement with male partners who may exploit their vulnerability and potentially take advantage of their access to children. This is dissimilar to assessment of risk and treatment focus for men who perpetrate CSA: “With men, we want them to be in a relationship with females, but for women, we want them to have the skills to step out of the relationship with men and stand on their own feet” (PP14).

The justice-involved participants mirrored the views of practitioners, with almost all indicating some level of partner influence related to their involvement in CSA. In line with the perspectives of practitioners, some of the justice-involved women indicated a direct and oppressive influence of their partners. For example, one participant said, “My partner very much had me under duress. He was bashing me” (JIP5). Other participants were less specific about their experiences of physical abuse, yet still indicated fear of retaliation from their partners for failure to comply: “My children’s biological father… was extremely abusive and controlling. I left my daughter with him for five days before I was able to get her. He threatened her life and his life” (JIP14).

Some of the justice-involved women explained their involvement with CSA was more a result of a fear of rejection and a history of relationship instability: “I have always been replaced very quickly… and I did what I could to not be replaced” (JIP9).

Many noted that challenges in their intimate relationships had filter-down effects on other relationships, including relationships with family, friends and colleagues. The majority reported that the controlling nature of their intimate partner relationships left them feeling apprehensive about the future, and their ability to trust others. One participant said, “I am scared to have a relationship again with a man and build trust, because my trust is already gone. I don’t have trust anymore. Even friendship. It’s gone” (JIP11).

Justice-involved participants explained that while they understood that their relationships were harmful, they were not ready to leave their partners, or did not know how to do this. Some explained that their self-esteem was so low that they did not feel they were worthy of a different partner. The justice-involved women in this study offered personal reflections and suggestions for how adverse relationship experiences should be addressed during treatment:

I’ve learned that empowerment for these women is a really big thing. I think these women need to know that it’s not a need for a man, it’s a want. I can’t deal with the rejection, but I need to recognize real acceptance (JIP9).

Many explained that direct advice from therapists/psychologists to leave unhealthy relationships was not well-received, and largely seen as patronizing. Instead, these women expressed a desire for practical support on how to navigate relationships, and assistance with building the necessary skills to handle conflict and identify toxicity in relationships.

### Theme 3: (Un)healthy Sexuality

Practitioners recommended treatment for women should include a component on (un)healthy sexuality. However, practitioners indicated that sexual motivations for CSA, while evident in some women, are less prevalent compared to men: “Women don’t tend to offend because they’re sexually motivated…It’s more male dominance that’s led them to where they are and to the behavior, which is quite different to the motivation for males” (PP3).

Addressing sexual deviancy in women was identified as a relevant treatment need by a minority of participants; the vast majority stated that this is not central to the treatment needs of many women, and too much emphasis on this may be counterproductive to treatment gains. Most suggested that treatment should instead focus on sexuality that is not directly related to the offense. For example, an individual’s triggers for promiscuity and perceptions of the role of sex in intimate relationships were identified as topics relevant for women, particularly when compared with treatment for men. Several practitioners gave examples from their own practice experience. They explained that women who perpetrate CSA often offend as a result of insecurity and feelings of low self-worth. This contributed to distorted views of gender, and beliefs regarding the role of women in pleasing a man. Others explained that sexual offending by women is a symptom of a larger issue with self-regulation, and that sexual interest in children is not often the primary driver of the offense for women: “More often than not…. the sexual behavior is just a behavior, especially for women. Therapy for distress tolerance and managing emotions would be better, instead of concentrating on the behavior” (PP2).

Similar to the observations of practitioners, none of the justice-involved women in this study identified that they had a sexual interest in children specifically. Most acknowledged wrongdoing and expressed regret for their actions, and many provided direct statements clarifying they “don’t think about children like that” (JIP12). Nevertheless, distortions were evident among some of the participants; some referred to underage girls as women or minimized and separated themselves from “real sex offender(s)” (JIP1, JIP12, JIP13). A small minority spoke about their difficulties in reading social cues and distortions surrounding children/adolescents whom they felt possessed adult-like qualities.

Despite some apparent distortions, assistance with addressing sexual interest in children was not a primary concern reported by any of the justice-involved women. In fact, the justice-involved participants in this study indicated that they would be unlikely to participate in a program if they felt they were labeled — or “treated like — a pedophile” (JIP6 & JIP13). Practitioners recommended that this topic should be addressed sensitively for women, and most indicated that, depending on the client, this topic could be integrated within other treatment targets instead (i.e., healthy relationships).

### Theme 4: Lowered Self-Concepts

The majority of practitioners indicated that low levels of self-esteem and self-worth are central to the causes of offending in many women, and expressed a need for more active consideration during treatment when compared with interventions for men: “For women, if we deal with her self-worth, her self-value and all of those underpinning things, then we *are* actually addressing her criminogenic needs, unlike doing that to a man”(PP2).

Many identified that low self-concepts not only impact on offending, but also heighten the risk of re-offending by inhibiting the desire to plan for a better future. Practitioners also explained that negative self-concepts might undermine engagement with rehabilitation and completion of programs, which may increase the risk of recidivism further. They suggested that this was often due to higher levels of shame in women, and that this also stems from a reluctance to have their own negative self-image confirmed by others. They offered suggestions for how this could be addressed, including variation in treatment content, format, and delivery, such as programs that contain both group and individual components, more intensive scheduling of treatment sessions, and secluded treatment spaces. Practitioners noted that motivational interviewing is common to many correctional programs, but was identified as being particularly important to treatment for women: “This is so important for women given their histories and all of the stigma and shame, which while similar to men, is different” (PP1).

The majority of justice-involved women also emphasized a need to address issues with lowered self-concepts. Several explained a desire for self-forgiveness, but also expressed doubt they could achieve this without significant therapeutic intervention. One participant said, “The more I talk about it, the more I hate myself for it. I have not forgiven myself, but I want to learn to do this” (JIP9). Many expressed high levels of anxiety about what their future might look like following release from custody; they believed that keeping busy and learning new skills would assist with building their sense of self and would help them move forward with planning for the future. Many suggested that a program that encompassed opportunities for skills-building, mindfulness, and structured communication would be useful to help break down some of their insecurities.

### Theme 5: Parenting Challenges

Practitioners noted that the greater likelihood of women being the primary caregiver of a child/ren presented additional challenges with reintegration when compared with men, often due to bureaucratic hurdles and stigmatization posing a barrier in pursuing child custody arrangements or maintaining contact with their children:

Many females have got children and they have to deal with child protection services treat them like s*** oops… Treat them badly…that adds to the burden of the situation, prevents them in many ways from going further because they're battling with, and trying to deal with, the stigmatization, rather than trying to deal with what they've done (PP21).

The need for general re-entry support was identified by almost all practitioners, with a primary focus on positive parenting and building skills and strategies to help maintain safe and strong parent-child relationships. Practitioners identified that this would be challenging given the restrictions of many women related to access to their children. However, they also noted that support is particularly important for women due to their greater likelihood of reunification with their children, and their potential to conceive and have more.

Over half of the justice-involved women were concerned that their relationship with their child/ren was strained as a result of their charges, and tension impacted other family relationships as well:

As a mum of course you worry and you want to have contact with your kids, but you can’t. You want to reach out and build a relationship with them again, but that won’t really work because they see you as a perpetrator, everyone sees you as a perpetrator. It’s really hard (JIP11).

Justice-involved participants in this study expressed disappointment with their experiences with government departments and decisions relating to their children, and identified that assistance with reunification and/or supervised communication with their children was desired:

There needs to be support away from the (relevant child safety department – de-identified for anonymity) because they don’t seem to help you. They seem to destroy your family. I want support with how to talk to ‘em, and try to get regular contact and stuff like that (JIP17).

It was identified by both participant groups that many women sentenced for CSA do not have long-term restrictions limiting all forms of contact with their children, so failure to strengthen parental capacity and give consideration to the needs of the child during treatment is likely disadvantageous.

Importantly, practitioners explained that the mental load of the separation challenges can also filter through to the children of these women and can cause them distress and alienation, which is counterproductive to treatment and long-term positive outcomes for the woman and her child/ren. One practitioner explained that:

Typically, there is not much consideration of the child of the offender in designing the program for the offender, which can do a lot of harm to the child. A program should link services together so that if there's some advantage to the child in retaining connection with their parent…and there can be a discussion about how this would best be managed (PP21).

Several practitioners were supportive of multi-systemic treatment options that were inclusive of the individual, community agencies, and the children and families of the client; when appropriately managed, many believed that this would be beneficial for all parties.

Both participant groups also noted that parenting support would assist in building client interest and motivation to participate in offense-specific programming, largely as it was future-focused and allowed these women to connect and set goals. A program that embedded components of positive parenting was also perceived as useful to re-invigorate clients following challenging or uncomfortable treatment sessions.

## Discussion

The current study incorporated the perceptions of practitioners and justice-involved women to learn more about the key considerations for gender-responsive correctional programming for women sentenced for CSA, particularly in Australia. A key finding of this research is that many treatment targets for women appear similar to the content of interventions available for men sentenced for CSA (Prenzler et al., 2023; Sousa et al., 2023), yet the needs of women are often expressed differently. This research found that gender-specific nuances appeared for five key areas, including the effects of past trauma, relationship toxicity, (un)healthy sexuality, lowered self-concepts, and parenting challenges. These findings help to inform how these topics are addressed when working with women. Importantly, and in alignment with other research findings, these treatment areas are interrelated and should be considered together within intervention planning, rather than as separate issues (Cortoni & Stefanov, 2020). However, this does not mean that all women with sexual convictions require the same intensity or type of offense-specific program, with some likely to benefit from more generalized programming instead (Hanson et al., 2025; Pflugradt et al., 2022). Largely, findings reflect other studies examining the preferred intervention targets and strategies for women sentenced for CSA, emphasizing the use of strength-based interventions focused on goal setting, independence, fulfillment, and stability (Cortoni, 2024; Pflugradt et al., 2018; Sousa et al., 2024).

Participants in this study reaffirmed the prevalence and impact of trauma, particularly gendered violence, in the histories of women who perpetrate CSA (Augarde & Rydon-Grange, 2022; Cortoni & Gannon, 2013). While men involved in CSA are also reported to have experiences with various forms of family dysfunction and childhood abuse (Levenson et al., 2015, 2016), the prevalence, source, frequency, and duration of trauma experiences make this an important gendered consideration for women. For men, the impact of childhood trauma experiences is often directed outward, resulting in a higher propensity to replicate the abuse, particularly when the perpetrator is a woman (Glasser et al., 2001). A history of trauma may manifest differently in women and may therefore require a more nuanced therapeutic response. This research supported the findings of others examining trauma histories in women’s offending pathways (e.g., Levenson et al., 2015; Pflugradt et al., 2018): women who have been victims of childhood sexual abuse often direct their trauma experiences inward, with the experience having a significant impact on their mental health and self-concepts (Glasser et al., 2001; Levenson et al., 2015; Neofytou, 2022). For women, these experiences appear to have less of a direct influence on their involvement in repeating the “victim-to-victimizer cycle”, with participants in this study describing a stronger impact on their relationships in adulthood, subsequently leading them to be involved in CSA perpetration (Glasser et al., 2001, p. 482). Many existing SOTPs for women provide opportunities for women to process trauma and its connection to offending pathways (e.g., Wanamaker et al., 2018). Building on this, a gendered trauma-informed approach to treatment will also require responsiveness to clinical features of women that may have manifested as a way to “navigate trauma” (Cortoni & Stefanov, 2020; Sousa et al., 2024: n/p). Practitioners in this study also reinforced the importance of program sequencing, which is consistent with other research examining the components of effective SOTPs for men (e.g., McKillop et al., 2022). Participants suggested that trauma-focused work should precede, but not replace, offense-specific intervention, to facilitate accountability and focus on criminogenic needs while enhancing responsivity and readiness for change (Sousa et al., 2024).

Relationship toxicity was reported to be a key characteristic of female involvement with CSA, often requiring a different treatment focus from men who perpetrate this crime. Both participant groups noted male influence, neglect, or a fear of men, were significant contributors to the motivations of many women involved in CSA (Beech et al., 2009; Gannon et al., 2012). Research on the influence of relationship instability in the etiology of men who perpetrate CSA shows a higher propensity to offend as a result of loneliness and fear of rejection, using CSA as a means to minimize these feelings (Marshall, 2010). While this is also apparent for some women, this was not found to be a direct motivation for the majority of women sentenced for CSA in this study, a finding that was also supported by practitioners. Consistent with earlier research (Marshall & Miller, 2020), this study found that involvement in adult relationships may influence risks of sexual offending in women. Both participant groups in this study indicated that, unlike men, many women sexually abuse children to maintain existing adult relationships, satisfy their partners, and mitigate the risk of future rejection and loneliness. Such dynamics align with Gannon and colleagues’ (2008, 2014) *directed avoidant* pathway, reinforcing the need for treatment approaches that target relational dependency to support autonomy and prosocial connection. Importantly, while relationships can play a positive role in desistance for both men and women (e.g., Farmer et al., 2015; Kendler et al., 2017), they may also expose some women to renewed risk when shaped by dependency or control. Indeed, this study found that women who perpetrate CSA can be obsequious in their relationships; therefore, to prevent “spiraling” or “reverting to script” (Bevan & Wehipeihana, 2015, p. 15), treatment for women should emphasize strength-based interventions encompassing tools for building self-efficacy, independence, and power (Sousa et al., 2024). This may then help women to have the confidence to pursue personal and social needs without the need for external validation. More support with building conflict resolution skills and healthy relationship management strategies would also be an important feature of responsive programming for these women.

Consistent with CSA offending typologies for women and other research exploring (un)healthy sexuality and/or sexual deviancy, sexual interest in children did not emerge as a central factor linked to offending for women in this study, or it was described as operating differently for women when compared with men (e.g., Blanchette & Taylor, 2010; Comartin et al., 2018). For example, both participant groups identified other complex sexual motivations, such as the role of sex in relationships and sex as a tool for self-validation, as potentially more relevant. However, the findings also reflect diversity in the offending profiles of women involved in CSA (e.g., Cortoni & Gannon, 2013; Pflugradt & Allen, 2015). As Gannon and Alleyne (2013) note, lone women may be more likely to hold offense-supportive beliefs directly related to their offending, whereas those engaged in co-offending may minimize such beliefs by attributing blame to their co-offender. Other research has found that sexual interests among women tend to be more fluid and relationally contextual (Chivers, 2017), and distortions, such as beliefs that children can consent or enjoy the experience, while less prevalent, may still require assessment and intervention where present (Blanchette & Taylor, 2010; Elliott et al., 2010; Pflugradt & Allen, 2015). Indeed, distortions were apparent within a small sample of justice-involved participants in this study, indicating a need to address such thinking in some cases (Gannon & Alleyne, 2013; Sousa et al., 2024). In terms of correctional programming, participants suggested that this topic should be addressed sensitively and assessed on an individual basis to avoid labels that may adversely impact program enrolment, participation, and completion. This will be necessary to support personal insight alongside accountability; a more subtle integration within broader program messaging could be considered by practitioners working with this population, with emphasis placed on strategies for maintaining healthy sexuality in future adult relationships.

Lowered self-concepts were identified as intrinsically linked to the core of women’s pathways into offending and an important treatment target to aid in crime desistance. A significant body of research reinforces the prevalence of mental illness, substance abuse, and harmful relationships in the lives of many women who perpetrate CSA (e.g., Augarde & Rydon-Grange, 2022), which may stem from, and be reinforced by, low self-esteem and self-confidence (Levenson et al., 2015). The participants in this study identified a need to rekindle and stabilize self-concepts, and suggested this may help twofold: it may increase openness and readiness to participate in intervention, and it may enhance the likely short-and-long-term impact of intervention. While accountability and adherence to evidence-based practice are important, offense-specific discussions will likely need to be approached with care so as not to further lower self-esteem and future outlook, which also applies when working with men sentenced for CSA (Marshall et al., 2009). However, particularly relevant for women, is that clinical intervention that focuses on lived experiences (and past traumas) was identified as a necessary focus of treatment, with some noting it is even preferential to forensic intervention concentrated on the offense itself. This finding aligns with existing research emphasizing women’s sexual offending as inseparable from broader life circumstances, relationships, and social contexts, requiring interventions that extend beyond offense-specific content, instead prioritizing more general criminogenic needs (Cortoni, 2024; Cortoni & Stefanov, 2020; Hanson et al., 2025; Pflugradt et al., 2022; Sousa et al., 2024). This is in contrast to many programs currently delivered to men, which often contain core modules on offense mapping and situational precipitators within relapse prevention (Prenzler et al., 2023; Sousa et al., 2023). Attention to the format and configuration of interventions is also necessary to assist in building social skills and self-confidence within this population; in particular, the more relational nature of many women, and the significance of social connection in building self-confidence, is an important treatment consideration for *how* programs should be delivered to women (Priebe et al., 2024b; Sousa et al., 2024).

Finally, an offense-specific program tailored to women sentenced for CSA should attend to some traditionally non-criminogenic or offense-related treatment areas, such as parenting support. This aligns with the recommendations outlined in the gendered treatment assessment framework proposed by Pflugradt et al. (2022), with particular attention given to the interconnection of needs for treatment responsivity among women who have sexually offended. While not currently a typical consideration for SOTPs for men, healthy parenting is a component of some existing SOTPs for women (Sousa et al., 2024), and participants in this study identified several benefits of addressing parenting concerns and capacity during treatment. For example, anxiety related to parental separation and uncertainty can be counterproductive to therapeutic gains as it serves as a distraction from working on other important program targets; thus, inclusion of parenting supports within programming could help to maintain interest and motivation for change (e.g., Pederson et al., 2024; Powell et al., 2017). In line with current programs and research on effective correctional practice, practitioners recommended that holistic interventions encompassing family involvement (when appropriate) may also help to improve readiness for custodial release and smooth transitional challenges; this was also a key suggestion of nearly all of the justice-involved participants. This strategy has been effective within interventions for juveniles involved in harmful sexual behaviors, where involvement of family in re-entry planning has been found “vital to the success of programs” (Calvert & Bauer, 2018, p. 8). Family involvement in treatment, and support with positive parenting strategies for women with sexual convictions, will likely require a complex facilitation process and management of potentially competing interests and needs. However, forming plans for the future and strengthening these relationships may ultimately serve as a vital informal control in their desistance from crime in the short- and long-term (Farmer et al., 2015).

## Conclusions, Limitations, and Future Directions

The current study utilized client and practitioner voices to enhance understanding of the key treatment targets for women sentenced for CSA. While there are apparent similarities with current correctional programs for men, gendered nuances were found, making it important to tailor the approach for women to ensure interventions are responsive to their unique needs. In line with current responses, there is a need for active attention to criminogenic factors to mitigate the risks of recidivism. However, programs should also address what women “are doing when they are not offending” (Lawson, 2008, p. 342). That is, a holistic, strength-based treatment approach for women sentenced for CSA is required, that includes program targets that are both criminogenic and non-criminogenic to address re-offending risk and the development of more pro-social behaviors (Cortoni, 2018; Cortoni & Stefanov, 2020; Lawson, 2008; Pederson et al., 2024). Programs need to be future-focused and designed to build confidence and competency in these women to desist from crime.

Importantly, participant suggestions for what to address during treatment show consistency with existing program targets within SOTPs for women internationally. However, further research, piloting, and implementation will be necessary to establish the effectiveness of such approaches and their suitability for different client demographics and offending profiles. Incorporating co-design strategies that include practitioner and client perspectives into program design, development, and evaluation will be vital moving forward, and will assist in the refinement of interventions to ensure they meet the needs and preferences of this cohort of women. Embedding these perspectives within a larger theoretically-driven and evidence-informed program design will ultimately help to enhance outcomes for women.

The findings of this research are significant for aiding the development of offense-specific correctional programming for women sentenced for CSA, particularly in Australia. Nevertheless, this study also has limitations. The results of this study are based on the subjective opinions of professionals and justice-involved women sentenced for CSA, which are likely shaped by their own experiences and personal biases. Moreover, although the findings align with many current international sexual offending programs for women, they are yet to be empirically tested with respect to their impact on engagement, treatment completion, and recidivism. While the sample of participants encompassed practitioners from a range of Western countries, and justice-involved women from three Australian states/territories, the scope of the research was limited to Western jurisdictions only; further research is needed to examine the suitability of suggestions within correctional programming in non-Westernized countries. Further research should also include First Nations perspectives, recognizing that cultural context will likely inform distinct treatment needs and experiences.

In spite of these limitations, this research provides further context for female-perpetrated CSA, and aids in the understanding of how to attend to the challenges that are most relevant to this population. Additional research into the utility of current correctional frameworks guiding practice, and the preferred modalities of treatment relevant to women is warranted.
